# Preoperative hypoalbuminemia and anemia as predictors of transfusion in radical nephrectomy for renal cell carcinoma: a retrospective study

**DOI:** 10.1186/s12871-015-0089-6

**Published:** 2015-07-21

**Authors:** Kyungmi Kim, Hyungseok Seo, Ji-Hyun Chin, Hyo-Jung Son, Jai-Hyun Hwang, Young-Kug Kim

**Affiliations:** Department of Anesthesiology and Pain Medicine, Asan Medical Center, University of Ulsan College of Medicine, 88 Olympic-ro 43-gil, Songpa-gu, Seoul 138-736 South Korea

**Keywords:** Anemia, Hypoalbuminemia, Radical nephrectomy, Renal cell carcinoma, Transfusion

## Abstract

**Background:**

The only curative therapy for renal cell carcinoma is the complete removal of malignant tissue. Surgical bleeding during radical nephrectomy may require blood transfusion. Blood transfusion, however, is associated with postoperative morbidity and mortality. This study investigated predictive factors of transfusion requirement in patients undergoing radical nephrectomy, as well as the effects of transfusion on postoperative outcomes.

**Methods:**

This study retrospectively enrolled 526 patients who underwent open radical nephrectomy for renal cell carcinoma between 2010 and 2012. Univariate and multivariate logistic regression analyses were used to determine independent predictive factors of a requirement for packed red blood cell (PRBC) transfusion. Postoperative outcomes included an admission to the intensive care unit (ICU) and lengths of ICU and hospital stay.

**Results:**

Of the 526 patients, 93 (17.7 %) required PRBC transfusion, with these patients requiring a mean 5.5 units. Preoperative hypoalbuminemia (serum albumin <3.5 g/dL) was observed in 75 (14.3 %) patients, and preoperative anemia (hemoglobin <12.0 g/dL) in 121 (23.0 %). Multivariate logistic regression analysis showed that preoperative hypoalbuminemia, preoperative anemia, and a high cancer stage were independent factors significantly associated with PRBC transfusion in open radical nephrectomy. The transfused group had higher incidence of ICU admission and longer lengths of ICU and hospital stay than the non-transfused group.

**Conclusions:**

Preoperative hypoalbuminemia and anemia are important predictors of PRBC transfusion during radical nephrectomy for renal cell carcinoma. Furthermore, transfusion is associated with poor postoperative outcomes.

## Background

Renal cell carcinoma, the most common form of kidney cancer, initially has an asymptomatic clinical course; thus, 25–30 % of patients present with metastatic disease at the time of diagnosis [1]. However, the increased utilization of abdominal radiologic imaging has led to an ever-growing proportion of incidentally discovered renal tumors. Since radical nephrectomy for localized renal masses results in excellent long-term prognosis, there have been steady advances in surgical techniques that have dramatically improved the ability to safely remove these tumors [2]. However, despite improvements in preoperative diagnostic modalities, anesthetic management, and perioperative care, the morbidity and mortality rates remain high, primarily due to hemorrhage and pulmonary embolism [3]. Therefore, it is important to identify factors predicting hemorrhage and subsequent blood transfusion, which may be associated with postoperative outcomes.

Most previous reports on nephrectomy have focused on the occurrence of postoperative complications [4–6]; however, information about factors predictive of intraoperative blood transfusion is limited. Therefore, the present study examined factors predictive of a requirement for packed red blood cell (PRBC) transfusion in patients undergoing open radical nephrectomy for renal cell carcinoma, as well as the effects of PRBC transfusion on postoperative outcomes after open radical nephrectomy.

## Methods

### Subjects

This study retrospectively reviewed the electronic medical records and laboratory results of all patients who underwent open radical nephrectomy for renal cell carcinoma at our institution between January 2010 and December 2012. We excluded the patients who underwent open partial nephrectomy, laparoscopic partial/radical nephrectomy or hand assisted laparoscopic surgery. The study protocol was approved by the Institutional Review Board of Asan Medical Center (AMC IRB-2013-0533).

During the study period, 548 patients with suspected kidney cancer underwent open radical nephrectomy. However, 6 patients who did not perform preoperative abdominal computed tomography (CT) and 4 patients with double primary renal cell carcinoma involving both kidneys were excluded. Furthermore, 12 patients with different pathologic diagnosis; 5 patients with metastatic renal nodule from lung or liver, 5 patients with ureter caner, 1 patient with transitional cell carcinoma in kidney and 1 patient with malignant mixed mullerian tumor, were excluded. Finally, a total of 526 patients were included. Demographic variables included patient age, gender, body mass index, history of hypertension, diabetes or abdominal surgery, coronary artery disease defined as stenosis of one or more coronary arteries with >50 % occlusion of the vessel lumen, and respiratory disease including chronic obstructive pulmonary disease, history of tuberculosis, asthma or interstitial lung disease. The standard preoperative evaluation at our institution included a clinical examination, chest X-ray, electrocardiography, complete blood counts, liver and renal function tests, coagulation profile, and viral serology. Renal cell carcinomas were staged based on preoperative imaging [7]. Nine patients without formal records of imaging findings did not have their cancer stage because they were diagnosed from other centers before visiting our center. Tumor size was also evaluated by abdominal CT.

### Anesthesia

Following the application of routine hemodynamic monitoring, general anesthesia was induced with thiopental, fentanyl, and rocuronium. After endotracheal intubation, patients were mechanically ventilated and their radial arterial pressure was monitored. Large bore intravenous lines were secured in all patients for fluid management. General anesthesia was maintained with volatile anesthetics, except in 14 patients who were maintained with total intravenous anesthesia, consisting of continuous target controlled infusion of propofol and remifentanil. Fluid management was performed using crystalloids and colloids. During anesthesia, patients were maintained at an arterial systolic blood pressure (SBP) of ≥90 mmHg and a hemoglobin concentration of ≥8.0 g/dL.

### Surgical procedure

Patients undergoing open radical nephrectomy with or without tumor thrombectomy were identified and their clinicopathological variables were recorded. Surgical incision was chosen based on an optimal approach to the tumor and vascular control. Open radical nephrectomy was performed in a standard manner by 4 urologists. Tumor thrombectomy was performed in 23 patients with venous thrombi. Five patients with a level IV tumor thrombus adherent to the right atrial wall also underwent cardiopulmonary bypass.

### PRBC transfusion criteria and postoperative outcomes

Intraoperative transfusion was performed in accordance with standard transfusion guidelines [8]. Indications for PRBC transfusion included an intraoperative hemoglobin concentration <8 g/dL. The incidence of intraoperative PRBC transfusion was determined from electronic medical records. Furthermore, creatinine levels which are the highest values within 3 postoperative days, pulmonary complications (such as fever >37.5 °C or atelectasis on chest X-ray, dyspnea, respiratory failure, and postoperative hypoxia up until 3 days after surgery) [9] and postoperative outcomes were evaluated. Postoperative outcomes included an admission to the intensive care unit (ICU) and lengths of ICU and hospital stay.

### Statistical analysis

Continuous variables were expressed as the mean ± standard deviation or number (percentage). Categorical data were analyzed using the *χ*^2^ or Fisher’s exact tests. Univariate logistic regression analysis was used to identify demographic, preoperative laboratory, and intraoperative factors significantly associated with PRBC transfusion requirement. Variables with a P value <0.05 in univariate analysis were included in the multivariate logistic regression analysis to determine independent factors predictive of PRBC transfusion requirement. A P value <0.05 was considered statistically significant. All statistical analyses were performed using SPSS 21.0 (SPSS Inc., Chicago, IL, USA).

## Results

The demographic characteristics, preoperative laboratory findings, and intraoperative variables of the 526 patients are shown Table 1. During open radical nephrectomy, 93 (17.7 %) patients received PRBC transfusions, with each receiving a mean 5.5 units of PRBCs. Preoperative hypoalbuminemia (serum albumin <3.5 g/dL) was observed in 75 (14.3 %) patients and preoperative anemia (hemoglobin <12.0 g/dL) in 121 (23.0 %).

**Table 1:** Characteristics, preoperative laboratory values, and intraoperative variables of patients who were and were not transfused with PRBC during radical nephrectomy

	Non-transfused group (*n* = 433)	Transfused group (*n* = 93)	*P* value
Age (years)	55.3 ± 12.8	57.6 ± 12.7	0.121
Gender (male/female)	277 (64.0 %) / 156 (36.0 %)	66 (70.9 %) / 27 (29.1 %)	0.199
Body mass index (kg/m^2^)	24.6 ± 3.4	23.6 ± 3.5	0.011
Hypertension	176 (40.6 %)	37 (39.8 %)	0.878
Diabetes mellitus	68 (15.7 %)	17 (18.3 %)	0.540
Coronary artery disease*	19 (4.4 %)	7 (7.5 %)	0.196
Respiratory disease^†^	25 (5.8 %)	9 (9.7 %)	0.167
History of abdominal operation	98 (22.6 %)	17 (18.3 %)	0.408
Preoperative laboratory values			
Albumin (g/dL)	4.1 ± 0.5	3.6 ± 0.7	<0.001
Albumin <3.5 g/dL	36 (8.3 %)	39 (41.9 %)	<0.001
Hemoglobin (g/dL)	13.5 ± 1.9	11.6 ± 2.2	0.009
Hemoglobin <12.0 g/dL	72 (16.6 %)	49 (52.7 %)	<0.001
Prothrombin time (INR)	0.99 ± 0.08	1.04 ± 0.10	<0.001
Platelet (×10^9^/L)	253.0 ± 78.1	293.8 ± 129.2	0.004
Creatinine (mg/dL)	1.3 ± 2.0	1.4 ± 2.3	0.965
Uric acid (mg/dL)	5.3 ± 1.5	5.3 ± 1.6	0.258
Crystalloid infused (mL)	1757.6 ± 760.4	3897.3 ± 2758.5	<0.001
Colloid infused (mL)	158.2 ± 268	1145.2 ± 861.9	<0.001
Operative time (min)	193.4 ± 57.7	332.5 ± 171	<0.001
Cancer stage^‡^			<0.001
I	239 (96.0 %)	10 (4.0 %)	
II	67 (82.7 %)	14 (17.3 %)	
III	85 (74.6 %)	29 (25.4 %)	
IV	38 (52.1 %)	35 (47.9 %)	
Urologist			0.570
A	154 (85.6 %)	26 (14.4 %)	
B	115 (80.4 %)	28 (19.6 %)	
C	73 (80.2 %)	18 (19.8 %)	
D	91 (81.3 %)	21 (18.8 %)	

Data are expressed as the mean ± standard deviation or number (percentage). ^*^Stenosis of one or more coronary arteries with occlusion of more than 50 % of the vessel lumen. ^†^Chronic obstructive pulmonary disease, history of tuberculosis, asthma or interstitial lung disease. ^‡^Based on preoperative imaging [7], except for nine patients without formal records of imaging findings. *PRBC* packed red blood cell; *INR* international normalized ratio

**Fig. 1:**
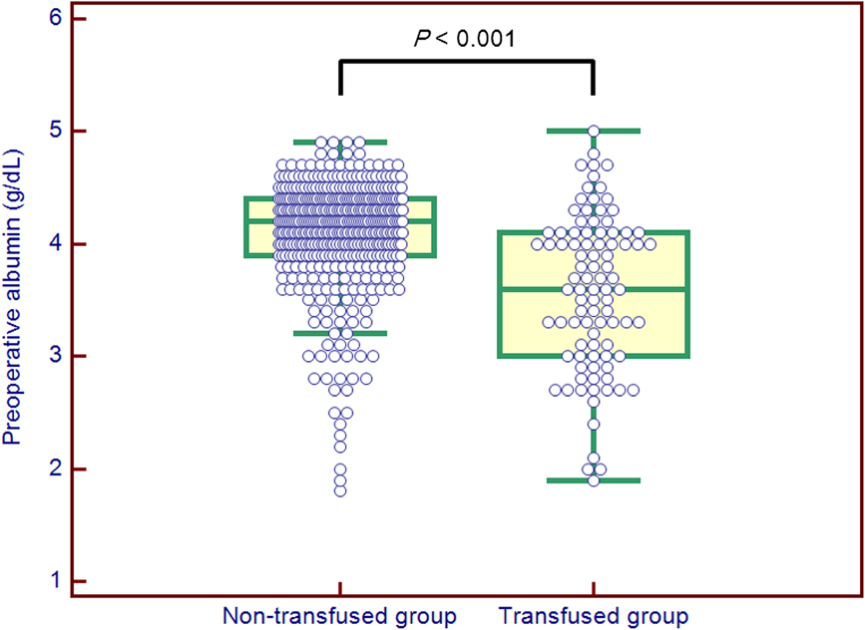
Box-and-Whisker plots of preoperative albumin levels in patients who were (transfused group) or were not (non-transfused group) transfused with PRBCs. Note that the preoperative albumin level was significantly lower in the transfused group than in the non-transfused group. PRBC: packed red blood cell

**Fig. 2:**
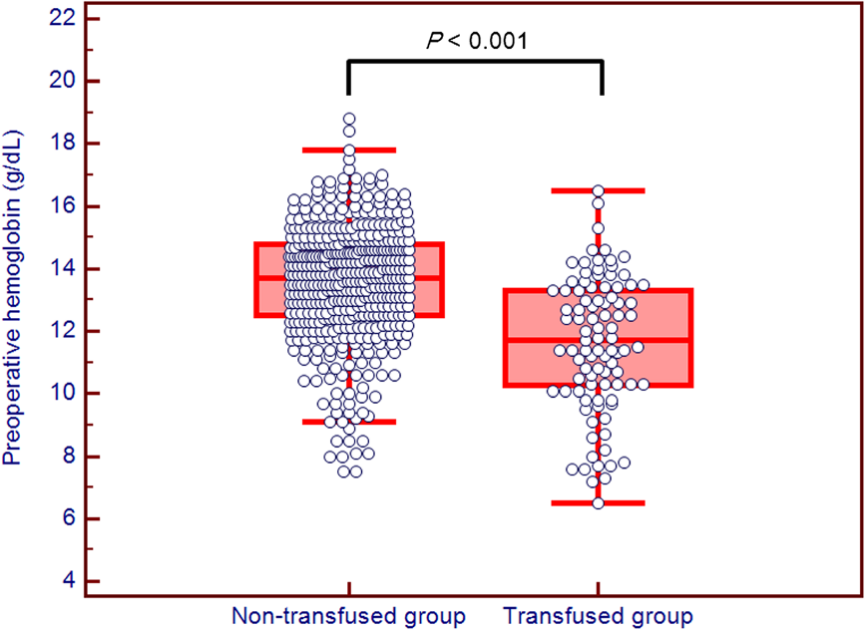
Box-and-Whisker plots of preoperative hemoglobin levels in patients who were (transfused group) or were not (non-transfused group) transfused with PRBCs. Note that the preoperative hemoglobin level was significantly lower in the transfused group than in the non-transfused group. PRBC: packed red blood cell

Comparisons between the 93 patients who required PRBC transfusions and the 433 who did not revealed no significant differences in demographic characteristics, except for body mass index (Table 1). However, preoperative serum albumin (Fig. 1) and hemoglobin (Fig. 2) concentrations were significantly lower in the transfused group than in the non-transfused group.

Univariate logistic regression analysis showed that body mass index, preoperative hypoalbuminemia, preoperative anemia, prothrombin time, platelet count, and cancer stage were significantly related to PRBC transfusion (Table 2). Multivariate logistic regression analysis showed that preoperative hypoalbuminemia, preoperative anemia, and more advanced cancer stage were independent factors significantly associated with PRBC transfusion in patients undergoing open radical nephrectomy for renal cell carcinoma (Table 2).

**Table 2:** Logistic regression analyses of factors independently predicting PRBC transfusion during radical nephrectomy

Variables	Univariate analysis		Multivariate analysis	
	Odds ratio (95 % CI)	*P* value	Odds ratio (95 % CI)	*P* value
Body mass index	0.919 (0.859 - 0.982)	0.013	1.014 (0.927 - 1.109)	0.764
Hypertension	0.965 (0.611 - 1.524)	0.878		
Diabetes mellitus	1.201 (0.668 - 2.158)	0.541		
Coronary artery disease^*^	1.769 (0.721 - 4.339)	0.213		
Respiratory disease^†^	1.749 (0.788 - 3.881)	0.170		
History of abdominal operation	0.765 (0.432 - 1.355)	0.358		
Hypoalbuminemia	7.965 (4.665 - 13.596)	<0.001	3.687 (1.758 - 7.736)	0.001
Anemia	5.584 (3.458 - 9.017)	<0.001	3.110 (1.635 - 5.918)	0.001
Prothrombin time	754.009 (62.390 - 9112.557)	<0.001	2.358 (0.083 - 67.249)	0.616
Platelet	1.004 (1.002 - 1.007)	<0.001	1.000 (0.997 - 1.003)	0.985
Cancer stage^‡^				
I	1.00		1.00	
II	4.994 (2.123 - 11.749)	<0.001	4.822 (1.950 - 11.920)	0.001
III	8.154 (3.813 - 17.439)	<0.001	9.431 (4.210 - 21.125)	<0.001
IV	22.013 (10.074 - 48.103)	<0.001	15.752 (6.835 - 36.302)	<0.001

^*^Stenosis of one or more coronary arteries with occlusion of more than 50 % of the vessel lumen. ^†^Chronic obstructive pulmonary disease, history of tuberculosis, asthma and interstitial lung disease. ^‡^Based on preoperative imaging [7]. *PRBC*, packed red blood cells

There was no significant difference in postoperative creatinine levels between non-transfused and transfused groups (1.62 ± 1.90 mg/dL vs. 1.80 ± 2.47 mg/dL, *P =* 0.507). However, the incidence of pulmonary complications was significantly higher for the transfused group than for the non-transfused group (32.3 % vs. 16.4 %, P <0.001). The rate of ICU admission was significantly higher and the lengths of ICU and hospital stay significantly longer for the transfused group than for the non-transfused group (Table 3).

**Table 3:** Postoperative outcomes of patients who were and were not transfused with PRBC during radical nephrectomy

	Non-transfused group (*n* = 433)	Transfused group (*n* = 93)	*P* value
ICU admission	8 (1.8 %)	34 (36.6 %)	<0.001
ICU stay (day)	0.06 ± 0.6	2.1 ± 8.4	<0.001
Hospital stay (day)	8.42 ± 3.0	16.9 ± 15.5	<0.001

Data are expressed as the mean ± standard deviation or number (percentage). *PRBC* packed red blood cells, *ICU* intensive care unit

## Discussion

The main finding of this study was that preoperative hypoalbuminemia, anemia and advanced cancer stage were independent predictors of increased PRBC transfusion requirements and subsequent poor postoperative outcomes in patients undergoing radical nephrectomy for renal cell carcinoma. To the best of our knowledge, this is the first report showing that preoperative hypoalbuminemia and anemia are important predictors of PRBC transfusion requirement in this group of patients.

Renal cell carcinoma accounts for 2 % to 3 % of malignancies in adults, as well as being the third most frequent and the most lethal form of genitourinary cancer [1, 10]. The only curative therapeutic strategy is the complete removal of malignant renal tissue [3]. Clavien-Dindo complications of grade II or higher have been recorded in 14.2 % of patients undergoing radical nephrectomy, including a requirement for blood transfusion in 11.1 % [11]. The findings presented here will provide important clinical information about perioperative management that may reduce transfusion-related morbidity in patients undergoing radical nephrectomy.

We found that preoperative hypoalbuminemia was an independent predictor of a requirement for PRBC transfusion during open radical nephrectomy. Serum albumin concentration is not only disease-specific but indirectly reflects the nutritional status of both acutely ill patients in the ICU and chronically ill patients with gastrointestinal tract bleeding [12–14]. Hypoalbuminemia may represent more advanced disease; moreover, because of the lower oncotic pressure may favor a leak of effective intravascular volume into the interstitial space, hypoalbuminemia may induce a hypovolemic state [15]. In patients with hypoalbuminemia and subsequent hypovolemia, the ensuing loss of blood during surgery can further reduce the effective circulating volume [16–18]. Hypoalbuminemic patients with reduced effective circulating volume are therefore more likely to require PRBC transfusion. Similarly, preoperative comorbid conditions associated with albumin concentrations <4.0 g/dL independently increase the need for blood transfusion [19]. Moreover, several studies using a modified version of the Clavien-Dindo classification system found that hypoalbuminemia was predictive factor of post-nephrectomy complications [20, 21]. Albumin level is also an independent predictor of massive blood transfusion in patients undergoing liver transplantation [22–25].

In agreement with results showing that preoperative anemia is a significant predictor of perioperative blood transfusion in patients undergoing elective surgery [26], the present study showed that preoperative anemia was significantly associated with a need for PRBC transfusion in patients undergoing open radical nephrectomy. These results suggest that patients with a low preoperative hemoglobin concentration reach a threshold for blood transfusion more rapidly than patients with a normal preoperative hemoglobin concentration [27, 28].

Blood transfusion has remained a mainstay for the management of severely anemic patients and of patients requiring a rapid increase in hemoglobin concentration [29]. Transfusion itself, however, carries significant risks and is associated with postoperative morbidity and mortality [30]. Similar to previous reports [30–32], we found that the group requiring PRBC transfusions were more likely to have poor postoperative outcomes, such as increased rate of postoperative ICU admission and longer ICU/hospital stay.

The present study found that advanced cancer stage was a predictor of the requirement of PRBC transfusion. Similarly, more advanced clinical T and N stages were significantly associated with higher rates of blood transfusion and postoperative complications [33]. Because of the level of tumor thrombus is included in T staging, it could also influence intraoperative blood loss and subsequent need for blood transfusion during surgery [34].

This study has a possible limitation. The present study was retrospectively designed, so we reviewed medical records for collecting data. However, the important perioperative information for the purpose of the present study is generally well documented.

## Conclusion

In conclusion, this study showed that preoperative hypoalbuminemia and anemia were important predictors of PRBC transfusion in patients undergoing radical nephrectomy for renal cell carcinoma. Moreover, blood transfusions were associated with poor postoperative outcomes. Identifying factors predictive of PRBC transfusion can provide better information about perioperative management, which may reduce the need for blood transfusion and, thus, prevent poor outcomes in patients undergoing radical nephrectomy.

## Abbreviations

CT: Computed tomography
ICU: Intensive care unit
INR: International normalized ratio
PRBC: Packed red blood cell
